# Supplementary material for: Are carbon emissions trading and green financial instruments synergistic? -Comprehensive quantitative research based on content analysis
Source: PLoS One. 2024 Mar 7;19(3):e0298601. doi: 10.1371/journal.pone.0298601 (PMC10919601; doi:10.1371/journal.pone.0298601)
Supplement: S1 Data — (ZIP) [file pone.0298601.s001.zip › Data/地级市Z_F工作报告.zip!/║╙─╧/┐¬╖Γ╩╨/└┤╫╘╣1⁄2╓┌║┼í╛┬φ┐╦╩2╛▌═°í┐_2014.docx]

各位代表：

　　现在，我代表市人民政府，向大会报告过去五年的政府工作，对今后五年及2014年工作提出建议，请予审议，并请各位政协委员和列席人员提出意见。

　　一、过去五年的工作

　　2009年以来，本届政府在省委、省政府和市委的正确领导下，在市人大、市政协的监督支持下，面对复杂的经济形势，积极作为，克难攻坚，全力打好“五大攻坚战”，较好地完成了市十三届人大确定的各项目标任务。全市生产总值从2008年的702.3亿元增加到2013年的1363.5亿元。规模以上工业增加值从191.4亿元增加到460.5亿元。固定资产投资从276.5亿元增加到941.7亿元。社会消费品零售总额从266.2亿元增加到577.8亿元。财政收入实现“183”目标，财政总收入从39.3亿元增加到103.9亿元，地方公共财政预算收入从26.1亿元增加到80.7亿元，市本级公共财政预算收入从11.1亿元增加到32.1亿元。城镇居民人均可支配收入和农民人均纯收入分别从11342元和4355元增加到19492元和8355元。2013年，规模以上工业增加值、固定资产投资、社会消费品零售总额、地方公共财政预算收入、第三产业增加值、城镇居民人均可支配收入增幅均居全省第1位。

　　五年来，我们主要做了以下工作：

　　（一）科学有效应对国际金融危机，经济保持快速健康发展。面对国际金融危机严重冲击，坚持加快发展不动摇，注重发挥工业、文化旅游业、扩大投资、金融支持、县域发展对全市经济的拉动作用，准确把握不同时段经济运行的突出矛盾和问题，先后出台促进经济平稳较快增长的一系列措施，开展了重点项目建设年、企业服务年、重点产业大招商等活动，全市经济发展速度连续五年超过全省平均水平，主要经济指标增幅持续位居全省前列。

　　（二）坚定不移实施工业强市战略，工业经济实力显著增强。全市规模以上工业企业达到1211家，比2008年增加446家，主营业务收入超10亿元企业达到24家。中阀科技、新大新材成功上市。积极培育主导产业，形成汽车（电动车）及零部件、装备、化工、新材料、食品、纺织服装、光伏、木业、生物医药、电子信息十大产业集群。重点项目建设实现新突破，五年来投资额以每年百亿元的速度递增，奇瑞汽车、瑞创农机、晋开集团、龙宇化工、横店东磁等一批重点工业项目建成投产，奇瑞集团、晋开集团主营业务收入分别突破40亿元和60亿元。按照“三规合一”、“四集一转”要求，规划建设8个产业集聚区，汴西、尉氏县、通许县产业集聚区分别被评为全省十强、十优、十快产业集聚区。

　　（三）着力打造经济社会发展核心增长极，开封新区建设及郑汴一体化发展取得新进展。开封经济开发区晋升为国家级经济技术开发区。金明区平稳并入开封新区。加大基础设施建设力度，完成投资118亿元，建成道路38条175公里，郑汴物流通道、郑开大道开封段拓宽、一至十二大街和十八大街等道路建成通车，新区启动区、铁南工业区基础设施进一步完善。中心商务区建设全面启动，迁村并居工程扎实推进，郑开大桥竣工通车，开封西湖基本完成开挖工程，一批中高档商住项目建成入住，学校、商贸广场等服务配套设施逐步完善，新区形象初步显现。主导产业培育力度加大，八个专业园区完成投资82.7亿元，新区入驻工业企业290家，汽车及零部件、农业机械、现代装备制造等主导产业初具规模。职业教育园区完成投资12.5亿元，入驻学校5所。港尉新区纳入郑州航空港经济综合实验区规划。郑汴一体化取得实质性进展，交通、金融、电信同城顺利实现。

　　（四）强力推进国际文化旅游名城建设，城市品位明显提升。开封被正式认定为“八朝古都”。宋都古城文化产业园区被确定为国家级文化产业示范园区。中国菊花文化节上升为国家级节会。连续五年成功举办“两节一论坛一赛事”，高标准举办第十届中国菊花展览会、第22届中国厨师节等重大文化旅游活动，荣获 “中国菊花名城”和全国首座“中国收藏文化名城”称号。新增全国重点文物保护单位6处。鼓楼复建、书店街改造、鼓楼街和寺后街街景整治、延庆观玉皇阁顶升、城墙保护四期、五期等一批文化旅游项目顺利完成，中原明珠文化旅游产业园、大宋文化博物馆·开封城摞城新郑门遗址、朱仙镇国家文化生态旅游示范区、清明上河城等一批文化产业项目启动实施，东京极地海洋馆建成开业，御河水系顺利通航，鼓楼街区荣膺 “中国著名商业街”称号，“一河两街三秀”成为开封文化旅游产业发展新亮点。完成龙亭湖景区周边综合整治，清明上河园荣膺国家5A级旅游景区。成功创建省级旅游标准化示范城市。全市旅游接待量、旅游综合收入分别从2529.4万人次和83.4亿元增加到4990.2万人次和207.8亿元，连续五年保持两位数增长。大力发展服务业，大润发综合购物广场、迪臣商业广场、开元商业广场、小宋城等建成开业，大商集团、金鑫珠宝等一批知名商业品牌入驻开封，商贸业态进一步提升。大力实施“万村千乡市场工程”，新建和改造农家店1552个，市场体系不断完善。 （上接第一版）成功引进浦发银行、交通银行。市商业银行在郑州设立支行。全市金融机构各项存贷款余额分别从442.7亿元和244.6亿元增加到1134.3亿元和699.4亿元。邮递、电信、住宿、餐饮、交通运输和房地产业发展层次进一步提升，第三产业增加值占生产总值比重连续五年居全省前列。

　　（五）统筹城乡发展，新型城镇化建设迈出新步伐。坚持高标准规划建设城市，五年编制完成各类专项规划、控制性详细规划370项，中心城市建成区面积由94.5平方公里扩展到124平方公里，城市框架进一步拉大。城市基础设施更加完善，复兴大道、金耀路、集英街、内顺城路等一批主次道路建成通车。大力实施“两改一建”，两年征收改造房屋面积709万平方米。数字化城市管理系统建成投用。依法整治规范市区三轮车，三轮车乱象得到根治。犬类治理成效明显。深入推进“三城联创”工作，成功创建省级文明城市，城市面貌显著改善。郑民高速开封段、连霍高速开封段拓宽工程建成通车，郑开城际铁路、郑徐客运专线、郑民高速二期、新开铁路、商登高速、机西高速等项目开封段开工建设。县城“三年百楼”和“十个一”工程基本完成，县城面貌焕然一新。新型农村社区建设扎实推进，开工建设112个，38个达到入住条件。扎实开展“双改”工作，43万农民转为城镇居民。加强农村基础设施建设，新改建农村公路2104公里，改造农村危房40377户，解决144.1万农村居民、在校师生安全饮水问题。完成扶贫开发整村推进村270个，26.4万农村贫困人口实现脱贫。

　　（六）提升农业产业化水平，农业基础进一步巩固。全面落实各项支农惠农政策，粮食直补、农资综合直补，家电、汽车（摩托车）下乡等惠农支出累计达到42.5亿元。实施高标准粮田“百千万”建设工程，粮食总产272.5万吨，实现“十连增”。提高农业机械化装备水平，启动精准农业与智能装备实验示范区建设，农业机械化率达到79.3%。大力发展菊花产业，菊花高新产业园启动建设。完成三义寨、柳园口、赵口灌区续建配套与节水改造等建设工程，新增改善灌溉除涝面积195万亩。实施现代农业产业化集群培育工程，省级农业产业化集群达到6个，省级以上农业产业化龙头企业达到20个。积极发展现代畜牧业，全市标准化养殖场达到970个，规模养殖场达到 2.5万个，畜牧业整体规模化经营达到50%以上。加快推进农业合作化产业化经营，各类农民专业合作组织发展到4468家。扎实推进林业生态市建设，完成造林73.5万亩。五县成功创建林业生态县。

　　（七）深入推进改革开放，发展动力和活力不断增强。企业改革成效显著，67家国有、集体企业改组、改制。鼓励、支持和引导非公有制经济发展，非公有制经济增加值占生产总值的比重达到62%。市县政府机构改革顺利实施。行政审批制度改革继续深化，五年精简行政审批事项247项。着力推进投融资体制改革，建立发投、城投、文投、新基投、交建五个市级投融资平台。医药卫生体制改革取得阶段性成果。国有文艺院团、文化市场综合执法等文化体制改革全面完成。警务机制改革、农村综合改革、集体林权制度改革成效明显。全部取消二级公路收费。深入开展大招商活动，在开封投资的世界及国内500强企业达到43家，五年利用外商直接投资12.4亿美元，引进省外资金1301亿元，进出口完成16.5亿美元，分别是上一个五年的6.3倍、3.5倍和2.7倍。加强对外交流与合作，与我市缔结友好关系的国外城市达到6个。

　　（八）着力促进经济社会可持续发展，发展方式加快转变。进一步提高科技创新能力，实施省级以上科技计划136项，省级以上工程技术研究中心、重点实验室和企业研发中心达到32家，建成博士后、院士工作站6个，国家高新技术企业达到25家。柔性引进两院院士、国家“千人计划”、著名高校等高层次人才40人。加强与中国农业大学、河南大学等院校合作，市校共建取得新成效。我市被评为全国科技进步先进市。扎实推进节能减排，实施建筑节能、公共机构节能等八大节能工程，加强重点排污企业监管，提升污水处理厂配套管网建设，全市化学需氧量、氨氮、二氧化硫排放量分别削减4%、5%和6.8%，完成节能减排目标。城市生活垃圾焚烧发电项目建成运营。实时发布PM2.5监测数据。加强环境保护和生态建设，全市出境河流断面水质考核指标达到省下目标，饮用水源地水质平均达标率保持100%。黄河滩区砖瓦窑厂全部关闭，全市完成土地整理42.8万亩，连续14年保持耕地总量占补平衡。

　　（九）更加关注和保障民生，幸福开封建设取得新成效。持续加大民生投入，连续五年办好为民实事，全市财政五年累计完成民生支出496亿元，占财政支出的68.2%。加强就业和社会保障工作，五年新增城镇就业38.1万人，农村劳动力新转移就业35万人以上。加大创业扶持力度，完成各类职业技能培训13.6万人，发放小额担保贷款38.1亿元。初步建立覆盖城乡的社会保障体系，城镇居民医保、城乡居民养老保险制度实现全覆盖，新农合参合率达到99.93%。企业退休人员养老金标准连年提高，城镇居民医保补助标准五年提高2.5倍，城乡低保、农村五保等社保对象待遇不断改善。积极发展老龄事业，养老机构床位达到5908张。市社会福利园区基本建成。落实法律援助，做到应援尽援。多层次住房保障体系初步形成，五年开工建设保障性住房5.06万套，基本建成3.2万套。大力推进文化惠民事业，一批标志性文化项目建成投用，刘青霞故居、市博物馆、汴京公园等一批文化设施免费开放，实现县县有图书馆、文化馆，乡乡有文化站，村村通广播电视、有农家书屋。五年来，全市教育支出从14.4亿元增加到38.7亿元，学前教育三年行动计划顺利完成，新建、改扩建幼儿园450所，城乡免费义务教育全面实施，中小学校舍安全工程完成预期目标。开封大学新校区基本建成，整合新建开封文化艺术职业学院。着力提高人民群众健康水平，卫生机构新增病床6570张，12类基本公共卫生服务免费向城乡居民提供，重大传染病防控救治成效明显。加强人口和计划生育工作，人口自然增长率连年低于全省平均水平。市人民体育场（馆）改造完成。全民健身运动广泛开展，竞技体育水平稳步提升。优先发展公共交通，更新城市公交车568辆，调整新增公交线路28条。妇女儿童、残疾人、慈善事业全面发展。城乡社区和基层自治组织服务功能进一步完善。以平安开封建设为主线，狠抓社会治安综合治理，严厉打击各类违法犯罪，切实加强信访稳定工作，信访形势总体平稳，安全生产、食品安全形势持续好转，社会大局保持和谐稳定。

　　五年来，我们自觉接受人大依法监督、政协民主监督和社会监督，认真办理人大议案、代表建议和政协提案5720件，满意率97.8%。深入推进依法治市和法制宣传教育，全社会法治意识不断增强。不断强化政府自身建设，持续推进“两转两提”，严格落实效能问责办法，政府职能和工作作风进一步转变，行政效能和公务员素质明显提高。坚持政务公开，强化行政监察和审计监督，扎实推进政府廉政建设。加强社会主义核心价值体系学习教育和精神文明创建，公民道德素质和社会文明程度持续提高。大力开展国防教育和国防后备力量建设，扎实做好“双拥”工作，开封驻军和武警部队在支持开封经济建设、维护社会稳定等方面作出了重要贡献，我市第六次荣获全国“双拥模范城”称号。民族宗教工作进一步加强。统计、外事、侨务、对台、档案、史志、地震、人防、气象、社科研究等工作都取得了新的成绩。

　　各位代表！

　　过去的五年，是开封历史上发展速度最快、城乡面貌变化最大、人民群众得到实惠最多的五年。五年的实践和探索，留下了许多宝贵经验和有益启示：一是始终坚持加快发展。把加快发展作为开封快速崛起的第一要务，坚持一张蓝图绘到底，用符合实际的发展思路指导工作，用行之有效的发展举措解决经济社会发展中的困难和问题，着力做大经济总量。二是始终坚持“五大攻坚战”。把工业作为强市之本，把文化旅游作为提升开封形象的新优势，把开封新区作为快速崛起的新希望，把新型城镇化作为改善开封面貌的新突破，把农业产业化作为县域经济发展的新亮点，心无旁骛，攻坚克难，强力推进。三是始终坚持改革开放。把改革创新和对外开放贯穿到经济社会发展的各个环节，以改革创新破除发展障碍，以扩大开放增强发展活力，进一步激发了经济社会发展动力。四是始终坚持抓好项目建设。坚持以大思路谋划大项目，大项目引领大投资，大投资促进大发展，正是一批重大项目的强力支撑，确保了我市发展速度不断加快、发展质量持续提升、经济实力显著增强。五是始终坚持改善民生。把发展经济与改善民生紧密结合起来，实实在在为人民群众办实事、谋福祉，不断提升人民群众的幸福感，让人民群众成为改革发展的现实受益者。六是始终坚持务实重干。面对困难不退缩，碰到矛盾不回避，踏实做事不浮夸，转变作风不松懈，凝神聚力，合力攻坚，营造了风清气正、求真务实的干事创业氛围。

　　五年的探索弥足珍贵，五年的成就鼓舞人心。这些成绩的取得，得益于省委、省政府和市委的正确领导，得益于市人大、市政协的监督支持，凝聚着全市人民的智慧和汗水。在此，我代表市人民政府，向全市工人、农民、知识分子、干部，向开封驻军指战员、武警官兵和公安民警，向各民主党派、工商联及社会各界人士致以崇高的敬意！向所有关心、支持开封发展的同志们、朋友们表示衷心的感谢！

　　在肯定成绩的同时，我们也清醒地认识到，我市经济社会发展中还存在不少困难和问题：一是综合实力不强，总量小、底子薄、基础弱、人均水平低的基本市情尚未根本改变。二是经济结构不优，二、三产业比重小，经济外向度低，加快经济结构调整、转变经济发展方式的任务还十分繁重。三是城镇化水平低，城乡公共服务和基础设施薄弱，城乡居民收入水平总体不高，统筹城乡发展任重道远。四是资金、土地、环保、能耗等制约因素日益突出，发展瓶颈亟待破解。五是公共卫生、文化教育欠账较多，收入分配、房屋征收、食品安全等一批人民群众关心关注的问题尚未得到很好解决，水污染、雾霾等环境问题日益突出，民生工作仍需继续努力。六是少数部门职能和作风转变还不到位，政府自身建设仍需继续加强。对此，我们一定高度重视，采取有效措施，认真加以解决。

　　二、今后五年的目标任务

　　过去五年，开封的发展成效全省瞩目；未来五年，开封的发展前景更加美好。省委九届六次全会提出，开封要向河南省新兴副中心城市迈进，为今后开封发展指明了方向并寄予厚望。未来五年，是我市转型升级、跨越赶超的关键时期，更是向全省新兴副中心城市迈进的重要阶段。国家全面深化改革、中原经济区战略实施、郑汴一体化发展、郑州航空港经济综合实验区建设等发展机遇叠加集聚开封，正在产生强大的综合效应，成为推动开封向全省新兴副中心城市迈进的强大动力。我市经过五年来的持续发展，生产总值突破1300亿元，财政总收入突破100亿元，综合竞争优势日益彰显，支撑发展体制机制日趋完备，为向全省新兴副中心城市迈进奠定了良好基础。全市上下政通人和，人心思进，发展自信越来越足，发展氛围越来越浓，建设全省新兴副中心城市的美好蓝图将进一步激发全市人民干事创业的豪情壮志，只要我们善于把握机遇，科学应对挑战，就一定能够赢得主动、赢得优势、赢得未来，谱写好迈向全省新兴副中心城市的锦绣篇章。

　　今后五年政府工作的总体要求是：认真贯彻落实党的十八大、十八届三中全会和习近平总书记系列讲话精神，坚持以邓小平理论、“三个代表”重要思想、科学发展观为指导，紧紧围绕建设经济文化强市总目标，把改革创新、扩大开放、转型升级贯穿到经济社会发展各个领域各个环节，抢抓中原经济区战略实施、郑汴一体化发展、郑州航空港经济综合实验区建设机遇，坚定不移打好“五大攻坚战”，努力建设实力开封、文化开封、美丽开封、幸福开封，加快向河南省新兴副中心城市迈进。

　　今后五年的预期目标是：

　　——实力开封建设迈上新台阶。生产总值年均增速高于全省平均水平，到2018年突破2000亿元；地方公共财政预算收入、全社会固定资产投资在2013年基础上翻一番，经济总量再上一个新台阶，辐射带动作用进一步增强。

　　——文化开封建设彰显新特色。文化软实力明显增强，文化事业和文化产业发展水平继续保持全省前列；文化特色更加鲜明，形成一批独具开封特色的文化产品；基本完成老城区改造，城市功能更加完善，“外在古典、内在时尚、宋韵彰显”的新宋风城市风格逐步形成；科教实力进一步提升，城市美誉度大幅提高。

　　——美丽开封建设展现新面貌。生态文明建设迈出坚实步伐，节能减排目标全面完成，水和大气质量明显改善，森林覆盖率达到22%，创建国家卫生城、文明城、园林城，建设天蓝地绿水净的美好家园。

　　——幸福开封建设取得新成效。城乡居民收入稳步增长，全市常住人口城镇化率达到52%，教育、医疗、文化等社会事业全面发展，就业、社保、医保、住房等社会保障体系更加健全，居民衣食住行用条件明显改善，社会更加和谐稳定，人民群众幸福指数显著提高。

　　三、2014年政府主要工作

　　2014年是全面贯彻落实党的十八大和十八届三中全会精神的重要一年，是完成 “十二五”规划目标的关键一年。中央提出坚持“稳中求进”的工作总基调，省委提出“调中求进、变中取胜、转中促好、改中激活”的总要求，根据我市经济社会发展实际和市委统一部署，今年政府工作要围绕“一个主题”，就是“瞄准高端，全面升级，努力打造经济文化强市”；把握“一个基调”，就是“好中求快”。在工作中，坚持高端策划，加快转型升级步伐，更加注重科学发展，更加注重解决制约经济社会发展的深层次问题，更加注重发展质量和效益，把快速增长、健康发展、质量效益同步提升的良好势头延续下去。

　　2014年全市经济社会发展的主要预期目标是：在转型升级、优化结构、提高质量效益的基础上，生产总值增长11%左右；地方公共财政预算收入增长15%；规模以上工业增加值增长17%左右；全社会固定资产投资增长22%左右；社会消费品零售总额增长15%；引进市外、省外资金增长20%，外贸出口总额增长12%；实际利用外资增长11%；城镇居民人均可支配收入和农民人均纯收入实际增长10%；城镇新增就业6万人以上；人口自然增长率控制在6.5‰以内；居民消费价格涨幅控制在3.5%以内；节能减排完成省下达任务。

　　为实现上述目标，重点抓好十个方面的工作：

　　（一）加快转型升级，打好工业强市攻坚战。坚持工业强市不动摇，突出工业在转型升级中的重要地位，强力实施工业强市“8155工程”和“五个一”攻坚行动，不断壮大工业经济实力，走科技含量高、经济效益好、资源消耗低、环境污染少、人力资源优势充分发挥的新型工业化道路。加快产业集聚区建设。围绕提升“四集一转”水平，加强基础设施建设，做大做强主导产业，拉长产业链条，力争产业集聚区完成固定资产投资和主营业务收入增长25%以上。加快产业转型升级。改造提升装备、化工、纺织服装等传统优势产业，大力发展汽车（电动车）及零部件、电子信息、新材料、生物医药等战略新兴产业，不断壮大产业规模，力争十大产业集群完成主营业务收入1850亿元，增长15%。加快培育主导产业骨干企业。大力开展服务企业活动，用足用好国家支持老工业基地城市建设政策，以50家重点骨干企业、50家高成长型企业为龙头，着力推动企业科技创新、产品升级，培育30家主营业务收入超10亿元的企业。加快重大工业项目建设。抓好晋开集团100万吨液体肥料60万吨复合肥、龙宇化工三期6万吨聚甲醛、LED液晶电视、智能手机、精细化工产业园等重大工业项目建设，带动全年工业投资超500亿元。

　　（二）做大做强服务业，打好建设国际文化旅游名城攻坚战。充分发挥文化旅游资源优势，坚持文商旅融合发展，着力做大做强服务业，加快国际文化旅游名城建设步伐。大力发展现代服务业。着力发展金融、文化、旅游、信息、电子商务等高成长性服务业，推动房地产、商贸流通、特色餐饮等传统服务业转型升级，培育发展健康服务、教育培训、商务服务、科技服务、养老及家庭服务等新兴服务业，提升现代物流、金融中介等生产性服务业水平，显著扩大产业规模和就业规模。坚持把中心商务区和特色商业区作为加快城区服务业发展的重要载体，实施双龙巷历史文化街区改造，建成七盛角民俗文化街区，规划建设开封汽车商贸城，努力建成一批大型城市综合体，培育一批金融服务、文化创意、商务中介、总部经济等特色楼宇经济，形成一批文商旅融合的特色商业街，改造一批现代物流、电子商务、会展等有机融合的专业市场。促进文化旅游产业快速发展。办好“两节一论坛一赛事”，提高“一河两街三秀”档次，高标准筹办好世界客属第27届恳亲大会。加快宋都古城文化产业示范园区建设，围绕“一城两环八区”空间布局，加快推进8大产业片区22个文化产业项目，抓好清明上河城、中原明珠文化旅游产业园、朱仙镇国家文化生态旅游示范区、大宋文化博物馆·开封城摞城新郑门遗址、刘青霞故居及汴京公园周边环境改造、艮岳宋代园林博物馆、客家文化交流中心和珠玑巷等项目建设。巩固省级旅游标准化示范城市成果，建设旅游集散中心，完善购物中心、星级酒店、停车场、旅游信息咨询网络体系等配套服务设施，做好御河、小宋城、皇宋大观文化园申报国家A级景区工作。启动黄河生态旅游规划，打造县域旅游新亮点，进一步拉长旅游产业链条。以“三城联创”为载体提升城市品位。深入开展“三城联创”工作，启动数字化城市管理系统二期工程，抓好农贸市场升级、早夜市整治、旱厕改造等薄弱环节，开工一批街心绿地、游园，严厉打击违法建设行为，加强城市交通秩序整治，强化城市精细化管理，着力提高市民文明素质，努力创建国家卫生城、园林城。

　　（三）加快推进郑汴融合，打好开封新区五年成规模攻坚战。主动对接，强力运作，打好开封新区五年成规模攻坚战。夯实发展基础，建设城乡一体化示范区。按照产城互动、综合配套的要求，重点推进启动区、铁南区、运粮河组团三大片区基础设施建设，同步完善供排水、供电、供气、供热、绿化、照明、污水处理等设施配套，确保完成基础设施投资30亿元以上。加快项目建设，培育新区发展新亮点。加快“八园一湖一中心”等137个重点项目建设，再筛选50个以上重大项目集中开工，带动全区完成固定资产投资200亿元以上。提高“八园”承载集聚服务功能，确保完成投资超过100亿元。快速推进开封西湖建设，力争早日蓄水成景。加快中心商务区项目建设，打造具有浓郁开封历史文化特色、古典时尚的中心商务区。进一步加大迁村并居工作力度，保障重大项目顺利实施。壮大主导产业，打造经济发展新引擎。加大招商引资力度，进一步壮大汽车及零部件、农业机械、空分设备三大制造基地规模，大力培育生物医药、电子信息、文化创意等战略性新兴产业，在产业升级和大项目上实现新进展。积极发展金融、商贸和物流产业，打造区域性金融、商贸和物流中心。深度融合，放大郑汴一体化战略优势。深入推进“五同城一共享”，深化交通同城，实现郑汴城际铁路开通运营。做好电信同城、金融同城后续工作，对接郑汴信贷同城业务，谋划开封金融后台服务中心。加快产业同城，增强两市在产业链、技术链、产品链方面的衔接，实现互补共赢。推动生态同城。积极协调文化、旅游、教育、科技等公共服务资源共享。加快港尉新区建设，积极融入郑州航空港经济综合实验区发展。

　　（四）科学推进城镇化，打好新型城镇化建设攻坚战。全面准确把握新型城镇化的内涵，坚持以人为本、传承文化、优化布局、产城互动、生态文明、科学有序的原则，着力构建“中心城市+县城+中心镇+新型农村社区”及“两核六组团”的新型城镇化发展格局。建好中心城市。统筹北区、东区、南区发展，完善规划，加快建设。严格执行城市总体规划，加大古城风貌保护力度，加快城区企业“退二进三”、“退城进园”，保护好传统街区，传承好古城文脉，着力打造“外在古典、内在时尚、宋韵彰显”的新宋风城市风格。实施城区“三贯通”工程，加快“两改一建”进度，市区完成棚户区城中村改造530万平方米。完善城市功能配套，启动机场北路、东平路等道路建设，完成迎宾路北延、新曹路、魏都路、文庙街、内顺城路、梁苑北路、夷山大街改造、官坊立交辅道及排水工程等城市基础设施建设，加快建设宋城路综合交通枢纽站，建成金明公交场站、仁和公交场站、城北旅游换乘站和新宋路污水提升泵站、汴京路雨水提升泵站。加快火车站周边开发改造。加强县城建设。坚持规划为引领、产业为支撑、商贸为载体、文化为灵魂、人居为根本，县城完成棚户区城中村改造220万平方米，启动实施规划提升、以产兴城等县城建设十大工程，提升县城品位，建设美丽宜居县城。发展中心镇。根据区位优势、自然生态、产业特色，着力抓好中心镇建设，不断增强中心镇辐射吸纳能力。完成市属乡镇总体规划修编，实施小城镇综合整治工程，提升小城镇整体发展水平。稳步推进新型农村社区建设。已建成的新型农村社区，建立健全可持续发展机制；已启动的新型农村社区加快建设，防止出现“半拉子”工程；未启动的新型农村社区，按照“五规合一”和“三集中”的要求稳妥推进。

　　（五）坚持做优农业，打好农业产业化提升攻坚战。坚持农业产业化道路，加快

　　土地流转，大力发展现代农业，促进农业生产经营专业化、标准化、规模化、集约化。继续实施高标准粮田“百千万”建设工程。持续推进粮食核心区建设，抓好高产万亩方、高标准粮田和四个百万亩优质特色农产品产业带建设，持续推进农业综合开发、土地整理和复垦开发，稳定提高农业综合生产能力，确保实现粮食“十一连丰”。继续实施现代农业产业化集群培育工程。大力培育农业产业集群，力争省级农业产业化集群达到8个以上，争取年收入超10亿元的产业化集群发展到15个。着力培育农业产业化龙头企业，重点扶持皓月、雏鹰、大用、天丰、禾丰、海壹食品等农业产业化龙头企业。加大农贸市场建设力度，再建10个高标准农产品批发市场。启动实施都市生态农业发展工程，在市区和县城周边，规划建设一批具有综合功能的农业生态园区。抓好水利基础设施建设。加强以抗旱除涝为重点的农田水利工程、引黄灌区续建配套与节水改造、中小河流治理、平原调蓄水库、病险水闸除险和小农水重点县等水利工程项目建设，完善引黄灌溉体系，确保新增改善有效灌溉面积30万亩以上，再解决34.9万农村居民、在校师生饮水安全问题。加快农业科技创新。抓好重大农业科技专项和重点科技计划项目实施，推进中国农业大学开封实验站、国家粮食加工装备工程技术研究中心、生物育种实验室、精准农业与智能装备实验示范区、晋开现代农业基地建设，促进农业科技成果转化和推广。加快推进基层农业技术推广服务体系建设，力争主要农作物良种覆盖率在99%以上。加强气象现代化建设，提升为农气象服务水平。构建新型农业经营体系。深化农村经营体制改革，培育专业大户、家庭农场、农民合作社、现代农业龙头企业等新型农业经营主体。加快农民专业合作社发展步伐，力争新培育200家农业专业合作社、10家农机专业合作社，新增土地流转40万亩。

　　（六）加强基础支撑建设，培育发展新优势。持续强化基础支撑服务体系建设，增强经济社会发展后劲，提升发展层次，拓展发展空间。强化基础设施支撑。推进“三条高速”、“三条铁路”、“一站一出口”建设，构建城区“五环九横十二纵”大交通体系。加快干线公路、县乡道路升级改造，提高县乡道路通行能力，打造市县间半小时交通圈。优化能源结构和布局，新建改造一批110千伏、220千伏输变电工程，推动农村电网改造升级。强化项目支撑。实施重点项目“358”工程，组织实施300个以上重点项目，重点推进“五大攻坚战”重大项目，带动全市重点项目投资突破800亿元。强化创新支撑。加强与国内外科研机构和创新基地的合作，鼓励和支持重点龙头骨干企业建立高水平技术研发中心，推进国家空分设备质量检测中心建设，新建省、市级工程技术研究中心8家以上。加大省级创新型（试点）企业和高新技术企业培育力度，新培育高新技术企业10家以上。强化人力资源支撑。大力实施创新型科技人才建设工程、全民技能振兴工程、技能人才回归工程等十大人才工程，盘活用好本地人才资源，把人口优势转化为人力资源优势。坚持引资与引智并重，加快实现由单纯项目招商向“人、财、项目”打包引进模式转变，力争引进两院院士、国家“千人计划”等高层次人才取得新进展。支持河南大学、黄河水利职业技术学院等高校发展，全力做好开封大学升本工作，办好开封文化艺术职业学院，切实提高办学质量和水平。大力发展职业教育，启动实施职业教育攻坚二期工程，争取入驻职教园区学校达到7所。强化信息化支撑。加快信息基础设施建设，实施“宽带开封”工程，推动4G技术规模商用，吸引知名企业在我市布局数据基地。采用先进信息技术改造提升传统产业，创建一批“两化”融合示范企业。加快电子商务发展，着力引进国内外知名电商入驻。提升城市信息系统综合应用水平，完成智慧城市顶层设计，加快智慧政务、智慧旅游、智慧交通、智慧医疗、智慧城管、智慧商务、智慧环保建设，实现市内公共服务区域WiFi全覆盖。

　　（七）加强生态环境建设，建设美丽开封。坚持节约资源与环境保护基本国策，加快建设天蓝地绿水净的美丽开封，增强可持续发展能力。以实施“四大工程”为重点改善环境质量。实施“蓝天工程”，落实好国家大气污染防治十条措施，推动重点行业、重点部位大气污染防治，有效遏制雾霾污染。实施“碧水工程”，抓好新一轮城镇污水处理厂和产业集聚区污水处理厂建设，开展现有污水处理厂提标改造工作，确保达标排放。深化惠济河综合整治，启动贾鲁河综合整治工程。加强饮用水源地保护，确保全市人民饮水安全。实施“清洁工程”，确保城市生活垃圾焚烧发电项目高效运营，实现城市垃圾无害化、资源化处理。加强农村面源污染治理，推进农村环境综合整治，开展美丽乡村建设，改善人居环境。实施“安全工程”，健全环境防范体系，实施好环保专项行动，保障环境安全。以加强生态系统建设为重点提升生态自然修复能力。完善生态补偿机制，实行最严格的源头保护制度、损害赔偿制度、责任追究制度。实施林业生态市提升工程，完成造林9.3万亩，加快高速公路、干线道路两侧绿色生态廊道建设。以加强总量减排为重点强化环境容量控制。强化工程减排、结构减排和管理减排，深化主要污染物排放总量预算管理，完善污染物排放许可证制度，积极开展排污权有偿使用和交易试点工作。建立项目环评审批与当地环境容量挂钩机制，加快淘汰落后产能。以成功创建国家新能源示范城市为契机，大力发展清洁能源，取缔集中供热及天然气管道覆盖范围内的小型燃煤锅炉。以节能降耗为重点提高资源节约集约利用水平。实施能源消费总量预算管理，建立能源承载能力监察预警机制，全面实施节能降耗行动计划，推行节能降耗技术改造和公共领域节能。加强再生资源回收网络体系建设，积极推动节能环保和再生产品消费，大力发展循环经济。

　　（八）全面深化改革，不断增强经济发展内生动力。加快推进一批看得准、有权限的改革，着力在重要领域和关键环节取得实质性进展。深化行政体制改革。加快事业单位分类改革。创新政府公共服务提供方式，研究出台政府向社会组织购买服务的指导意见。推进工商注册制度便利化。推动交通运输管理体制和行政执法体制改革。改革完善食品药品监管体制。激发市场主体活力。坚持市场在资源配置中的决定性作用，深化企业内部制度改革，完善现代企业制度和法人治理结构，加强国有资产监管。支持非公有制经济健康发展，完善企业服务机制、政企沟通机制，及时解决民营企业和中小企业发展中的问题，鼓励非公有制企业参与国有企业改革。大力发展混合所有制经济。扎实推进财税改革。完善预算体系，推进预算公开。清理规范税收优惠政策，全面落实国家“营改增”等改革政策。加强预算绩效管理，盘活财政存量资金。加强政府债务管理，有效防范和化解财政风险。推动投融资平台整合功能，提升投融资能力、资产管理能力和风险防范能力。深化金融改革。充分利用多层次资本市场、多种金融产品，推进企业上市融资。推动符合条件的县域农信社组建农商行，加快村镇银行组建步伐，努力实现村镇银行县域全覆盖。加大金融机构引进力度，健全金融体系。支持具备条件的民间资本依法发起设立金融机构。加强金融服务公司建设，为企业提供优质金融服务。建立风险补偿机制，优化小微企业融资环境。推进资源性产品价格改革。全面实施居民生活阶梯水价，加大差别电价、惩罚性电价实施力度，理顺天然气与可替代能源比价关系。推进大用户直购电试点、煤电价格联动机制改革，基本完成农电体制改革。深化土地管理制度改革。完成农村宅基地确权登记和颁证工作，探索宅基地有偿退出机制，积极争取开展宅基地制度改革试点。推进通许县农村土地承包经营权登记试点工作。 （上接第二版）探索建立兼顾国家、集体、个人的土地增值收益分配机制，研究在符合规划和用途管制前提下，开展农村集体经营性建设用地进入市场流转试点。完善人地挂钩试点政策，拓展县域经济发展用地空间。探索实行租让结合、分阶段出让的工业用地供应制度。

　　（九）持续扩大对外开放，提高开放型经济水平。把2014年确定为对外开放提升年，持续推进重点产业大招商，围绕十大产业集群，瞄准短板，拉长链条，创新招商方式，确保对外开放工作实现新突破。注重招大引强。坚持把“招大引强”作为当前和今后一个时期招商引资工作的重点，瞄准国内外500强、民营企业500强、央企和大型跨国公司、行业龙头企业，争取每个产业、每个产业集聚区引进、落户2~3个投资10亿元以上的战略支撑项目或龙头企业，每个县区争取引进1~2个投资2000万美元以上的外资企业。注重招优引新。立足转型升级，瞄准“高质、高新、高端”，更加侧重新兴产业和延长产业链条项目招商，围绕汽车（电动车）及零部件、电子信息、智能终端、生物医药、新材料、高端化工、航空物流、新型服务业等新兴产业，开展重点招商，加快推动产业结构转型升级。推动电动车、纺织、汽车及零部件产业基地争创省级出口基地，扩大对外贸易竞争优势。加大对利用外资的考核奖励力度，切实提升全市经济外向度。注重拓宽领域。抓住制约经济社会发展的关键环节，围绕文化旅游、两改一建、老城区改造、县城及“三区”建设、“三贯通”工程、社会事业、职业教育等关键领域，制定针对性招商方案，全力实施，重点突破。注重方式创新。突出专业化集群式招商，更加注重区域招商、中介招商、以商招商，更加注重发挥企业在招商引资中的主体作用和商会、协会的桥梁作用，积极探索“全链条、全要素、全服务、无障碍”的立体招商模式，走出一条招商引资新路子。

　　（十）切实保障和改善民生，建设幸福开封。把保障和改善民生作为政府工作的根本出发点和落脚点，继续加大民生投入，实施好“十项重点民生工程”。千方百计扩大就业。实施更加积极的就业政策，健全公共就业服务体系，重点做好高校毕业生、农村转移劳动力、下岗失业人员、退役军人就业工作，确保新增城镇就业6万人。加大困难就业群体帮扶力度，推动实现更高质量就业。切实加强社会保障。继续扩大社会保障覆盖范围，加强城镇职工医保、城镇居民医保、新农合制度建设。改革创新社保经办机构。进一步完善失业、医疗、工伤、生育保险市级统筹，建立待遇正常调整增长机制。进一步完善社会救助制度，不断提高救助标准。加大农村扶贫开发，完成30个贫困村整村推进任务，解决4万农村贫困人口脱贫问题。强力推进保障性安居工程建设。开工建设公租房3202套、棚户区城中村改造安置住房9022套，完善保障房保障机制，着力缓解中低收入群体住房困难。持续改善居民生活条件。提升水、电、燃气、供热等公共服务水平，新增1200万立方米清洁能源，市区新增集中供热面积200万平方米。加快便民利民设施建设，再建设200家放心馒头店、50家便民菜店，建成一批公共体育设施。大力发展公共交通，进一步改善出行条件。加强居民小区物业管理工作，改善居住环境。努力办好人民满意的教育。坚持教育优先发展战略，深化教育领域综合改革，全面实施学校精细化管理。不断加大教育投入，积极鼓励民间资本兴办教育。巩固学前教育三年行动计划成果，提高办园水平。推进义务教育均衡发展，抓好中小学布局调整、义务教育学校标准化建设、农村义务教育学校改造，推动普通高中教育内涵发展。支持民族教育和特殊教育发展。提高医疗卫生保障水平。巩固基本医疗保障制度，全面推广县级公立医院新农合按病种付费工作。深入实施基本药物制度，实现基层医疗卫生机构基本药物制度全覆盖，提高基本药物配送率。加快基层医疗卫生机构综合改革，继续深入做好基本公共卫生服务和重大公共卫生服务项目，加强医疗急救网络体系建设，推进我市全科医生临床培养基地、中心医院、妇儿医院等重大卫生项目建设。支持中医药事业发展。抓好重大传染病防控。有序推进生育政策调整，促进人口长期均衡发展。健全市福利园区服务体系，加快县区福利中心建设，完善弱势群体保障功能。大力发展养老产业，积极发展社区居家养老服务，提高养老服务水平。扩大基本公共文化服务。支持市图书馆、市博物馆、市美术馆、市群艺馆等公共文化基础设施建设，扶持民间博物馆发展，不断充实和丰富公共文化服务内容。实施文化体育惠民工程，广泛开展全民健身运动，提高竞技体育水平，启动市体育中心建设，精心组织“舞台艺术送农民”活动、政府采购公益性演出活动、农村电影放映工程，继续推进公益性文化设施免费开放。大力倡导全民读书。加强和创新社会治理。以平安开封建设为主线，深化安全生产管理体制改革，强化安全生产，坚决遏制重特大事故发生。提升食品药品安全监管水平，确保广大人民群众“舌尖上的安全”。加强诚信体系建设，发挥中介组织在社会管理中的作用。积极推进社区矫正和人民调解工作。扎实开展“六五”普法和法律援助工作。强化信访工作和社会治安综合治理，依法打击各种违法犯罪活动，确保社会大局和谐稳定。

　　继续加强国防动员、国防教育和国防后备力量建设，支持开封驻军、武警部队、预备役部队、民兵和人民防空建设，深入开展双拥共建，促进军地融合发展。支持工会、共青团、妇联、工商联等人民团体发挥桥梁纽带作用。全面贯彻党的民族宗教政策，依法管理民族宗教事务。认真做好第三次经济普查。继续做好外事、侨务、对台、史志、地震、社科研究等工作。

　　四、加强政府自身建设

　　把依法行政、转变职能、作风建设、廉政建设作为政府自身建设的关键，深入推进“两转两提”，不断提高政府工作水平。

　　（一）加快推进法治型政府建设。坚持依法治市，严格按照法定权限和程序履行职责，不断提高政府公信力和执行力。严格执行人大及其常委会决议决定，自觉接受法律和工作监督，主动接受人民政协民主监督，支持政协参政议政，扎实办理人大代表建议和政协提案，广泛听取民主党派、无党派人士和人民团体意见建议。强化政务信息公开，加强政府网站、市长专线建设，自觉接受社会监督和舆论监督。坚持科学民主依法决策，完善决策程序，健全合法性审查、集体讨论、专家论证、社会公示和听证、决策责任追究等制度。加强行政行为的规范和监督，确保严格执法、公正执法、文明执法，努力让老百姓在每一个行政执法行为中都能感受到公平和正义。

　　（二）加快推进服务型政府建设。加快政府职能转变，加强制度建设，推动政府职能向创造良好发展环境、提供优质公共服务、维护社会公平正义转变，努力实现好、维护好、发展好人民群众的根本利益。深入开展学习型机关创建，完善公务员培训、考核、奖惩机制，加强公务员队伍建设，不断提高为人民服务的能力和水平。开展好第二批党的群众路线教育实践活动，从群众反映最强烈的问题改起，从群众意见最集中的地方抓起，从群众最关心的事情干起，努力提供优质、均等、便捷的公共服务，让老百姓生活得更加幸福、更有尊严。

　　（三）加快推进效能型政府建设。继续开展效能建设年工作，不断完善效能问责办法，加大效能问责力度，进一步提高行政效能。以深化改革为契机，继续减少和下放一批审批事项，简化办事程序，创新审批方式，提高审批效率。强化行政服务中心功能，建成市民之家，建立部门审批事项目录清单制度并向社会公开，巩固完善“一个窗口集中办理”、“一站式办成”、“限时办结”的行政审批模式，持之以恒开展贴心服务、主动服务、承诺服务，千方百计让老百姓办事更加方便快捷、满意舒心。

　　（四）加快推进廉洁型政府建设。认真落实中央八项规定，严格执行党风廉政建设责任制，全面推进惩治和预防腐败体系建设。严格规范权力行使，健全权力运行制约和监督机制。完善目标考核评价办法，树立正确的考核导向。加强廉政监察和审计监督。加大违纪违法案件查处力度，严肃惩治不正之风和腐败行为。严格落实《党政机关厉行节约反对浪费条例》等规定，健全厉行节约反对浪费制度体系，确保“三公”经费只减不增，禁止新建政府性楼堂馆所。政府过紧日子，就是要让老百姓过上好日子。

　　各位代表！

　　打造河南省新兴副中心城市和经济文化强市的宏伟事业鼓舞着我们，全市人民的热切期望和殷殷重托激励着我们。让我们高举中国特色社会主义伟大旗帜，紧密团结在以习近平同志为总书记的党中央周围，在省委、省政府和市委的坚强领导下，解放思想，开拓创新，务实重干，克难攻坚，为早日把开封建成河南省新兴副中心城市和经济文化强市而努力奋斗！
